# Gene Therapy Evidence Generation and Economic Analysis: Pragmatic Considerations to Facilitate Fit-for-Purpose Health Technology Assessment

**DOI:** 10.3389/fpubh.2022.773629

**Published:** 2022-02-09

**Authors:** Tingting Qiu, Michal Pochopien, Shuyao Liang, Gauri Saal, Ewelina Paterak, Justyna Janik, Mondher Toumi

**Affiliations:** ^1^Département de Santé Publique, Aix-Marseille Université, Marseille, France; ^2^Department of Health Economics and Outcomes Research, Creativ-Ceutical, Warsaw, Poland; ^3^Department of Health Economics and Outcomes Research, Apothecom, London, United Kingdom

**Keywords:** gene therapies, health technology assessment, economic analysis, recommendations, affordability

## Abstract

Gene therapies (GTs) are considered to be a paradigm-shifting class of treatments with the potential to treat previously incurable diseases or those with significant unmet treatment needs. However, considerable challenges remain in their health technology assessment (HTA), mainly stemming from the inability to perform robust clinical trials to convince decision-makers to pay the high prices for the potential long-term treatment benefits provided. This article aims to review the recommendations that have been published for evidence generation and economic analysis for GTs against the feasibility of their implementation within current HTA decision analysis frameworks. After reviewing the systematically identified literature, we found that questions remain on the appropriateness of GT evidence generation, considering that additional, broader values brought by GTs seem insufficiently incorporated within proposed analytic methods. In cases where innovative methods are proposed, HTA organizations remain highly conservative and resistant to change their reference case and decision analysis framework. Such resistances are largely attributed to the substantial evidence uncertainty, resource-consuming administration process, and the absence of consensus on the optimized methodology to balance all the advantages and potential pitfalls of GTs.

## Introduction

Gene therapies (GTs) have shown extraordinary promise for the mitigation or potential cure of a broad spectrum of life-threatening or debilitating diseases, such as cancers and retinal disorders (1). Yet, despite the innovative treatment paradigm and exceptional clinical benefits potentially provided by some GTs (2, 3), numerous obstacles to efficient market access prevail (4). These obstacles range from resource-consuming manufacturing processes to reimbursement and funding challenges (4–8). In two key instances, the sum of these obstacles has even proven to be unmanageable, and as such, the GTs has been withdrawn from the market (4, 9).

In particular, the limited clinical evidence and high up-front treatment costs for GTs have challenged their evaluations in health technology assessment (HTA); hence, specific considerations have been needed. Recommendations to mitigate such uncertainties in payer decision-making of GTs continue to be published since the first GT, alipogene tiparvovec (Glybera^®^), was approved in Europe in 2012 (10). However, the feasibility of integrating the evidence generation approaches into existing HTA guidelines or decision analysis frameworks (DAFs) remains unclear. The applicability of these recommendations and the utility of the existing evidence could be limited, therefore delaying the inclusion of the GTs into health system formularies and thus patient access (11). To this end, we have reviewed the published recommendations for evidence generation and economic analysis for GTs, with an aim to critically explore the feasibility of implementing the recommended approaches within current HTA DAFs (Table 1).

**Table 1:** Recommendations from publications and our perspectives.

**Subjects**	**Recommendation for economic analysis of GTs**	**Comments on feasibility of implementations**
**RECOMMENDATIONS TO MITIGATE THE UNCERTAINTIES IN THE CLINICAL TRIALS**		
Surrogate endpoints	It is important to determine the appropriate approaches for the measurements and validation of surrogate endpoints to final clinical endpoints, e.g., mortality, survival, important clinical events or health-related quality of life (HRQoL) (22–24).	It may be difficult for manufacturers if such surrogate endpoints could not be identified in databases or in registries. This may often not address HTA requirement.
	The relationship between surrogate endpoints and final clinical endpoints could be examined from the aspects of biological plausibility, cohort levels, clinical trials or systematic review (20, 23, 25).	Although this is good practice to raise these questions for any research finding in life science, it only supports the appreciation of the potential relationships between outcome and intervention. However, it will not be considered by HTA as pivotal evidence but supportive evidence. Payers defined clear methodology to validate surrogate endpoints. Unless such methodology is followed, the validation may not be accepted by most HTA
Evidence besides single-arm trials	The rationales behind conducting non-comparative studies should be clearly provided. This could include but not limited to that comparative studies can increase the risk of irreversible damage, delaying access to poorly serviced patients, difficulty in patient recruitment from very small populations, etc. (17).	Although this helps to justify why a comparative trial was not feasible, it does not change the fact that the evidence is difficult to assess when comparative data is not available.
	The inclusion of non-randomized data to provide important information complementary to single-arm trial, for example, natural history studies, observational studies, patient registry database or medical chart to serve as historical control (20, 22–25).	Historical control study is rarely accepted by HTA bodies to make decisions.
	Network meta-analysis and multivariate meta-analysis could also be used to measure comparative effectiveness and reduce the uncertainty around the effect estimates (20, 23).	This is always possible but such analysis, when only single arm study is available for the new intervention, is associated to methodological challenges and high uncertainty.
	The bias of historical control group could be minimized if the magnitude of treatment benefits is dramatic, the primary endpoint is objective, the heterogeneity in patient population and study outcomes is explored, and the confounding factors were well-known and adequately adjusted with suitable statistical analysis methods (17, 23, 26, 27).	The generalizability and transferability of the clinical data toward the historical cohort were considered. When all these criteria are met, this significantly increases the chances to have HTA accepting the outcome of single arm trials.
Quantifying uncertainty	Post-launch real world evidence (RWE) collection is critical to confirm the treatment benefits and bridge the evidence gaps in the initial submission (19, 26, 36–38).	However, it is likely that payers will not be willing to pay high prices until the long-term evidence is available as in the case of gene therapies long-term follow up studies are always required
	Coordination across countries and isolated private manufacturers should be encouraged to enhance greater consistency and efficiency of RWE collection (19, 23).	This will help reaching conclusion faster with more powerful conclusions because of the consolidation of evidence collected from multiple countries, simultaneously. However, it does not address the question related to the non-comparative study design. Moreover, depending on country-specific restrictions to reimbursement, it may not be acceptable to pool multi-countries data.
	Sensitivity analysis (e.g., probabilistic or deterministic) and/or scenario analysis to measure the impact of model assumption and input parameters, to examine the robustness of study result and to quantify the uncertainties of the study, such as drug cost, comparators used, different data sources, different time horizon (i.e., long-term and short-term) and different treatment benefits estimates (e.g., optimistic and conservative benefits scenarios) (17, 20, 22, 24–27).	The most critical uncertainty for GT is the durability of effect and potential long-term safety. In most cases of GTs, durability of effectiveness is flat, making impossible to extrapolate the efficacy using the traditional or specific method to account for the flattening of the tail of the survival curve. Hence, the sensitivity analysis even using scenario does not address the most critical uncertainty from HTA perspective
	Value of information (VOI) analysis as an adjunct to HTA could be employed to explore the evidence uncertainty and inform the further evidence collection (22, 24, 26).	Value of information allow to assess if performing an additional study will bring valuable information, but does not help HTA decision-making at time of launch
	Interactions with other relevant stakeholders including patients, clinicians, experts (e.g., medical scientists, statisticians, economics professionals), regulators, HTA bodies and payers to understand the varying interests of each party (19, 23, 36, 38).	This will not address HTA perspective on uncertainty at the time of launch.
**RECOMMENDATIONS TO CONDUCT ECONOMIC ANALYSIS**		
Calculation of total cost	Apart from the acquisition price, more in-depth considerations of all costs and resources that are required to provide GTs should be taken in total cost calculation, which could include but not limit to: additional infrastructure cost on the healthcare system, cost for managing adverse events, expenses for patients to traveling to specialized medical centers (20, 22, 26, 27).	This is a prerequisite of a robust HTA, but it may be difficult to comprehensively assess such cost due to lack of reliable data
Value assessment	Novel value elements, that beyond the direct health benefits but relevant to patients, caregivers and whole society, are worthy of considerations when performing the value assessment of GTs (17, 19, 22–25, 31, 37, 42).	Although this is very relevant for HTA to consider both payer and society perspective, the switch from payer perspective to society perspective will not happen in short, medium term. Likewise, novel elements of value were often described in publications, but have limited impacts on HTA bodies which concentrate their attentions mainly on effectiveness and cost effectiveness
	These factors could be grouped into 3 classes as disease-related values, indirect values, and other broader values (17, 19, 22–25, 31, 37, 42).	This is usually not considered by most HTA bodies, and therefore it is unlikely that these HTA bodies will change their assessment framework for GTs.
	Cost-effectiveness analysis is recommended to provide two references cases analysis from both healthcare sector and societal perspective (17, 22, 23, 25, 26).	Countries will continue their practices of applying payer perspective, and will not change their operations in the short term, despite it makes a lot of sense to consider a society perspective in case of GTs
	Using multiple criteria decision analysis (MCDA) to enable the incorporation of additional values as well as their relative weightiness in a deliberate way (22, 23, 26).	The experience of NICE to consider MCDA was negative. So far, no HTA bodies consider MCDA, mainly because of methodological reasons related to elicitation of the weight for attribute and the definition of threshold. Some experts considered that cost effectiveness could not be an attribute for MCDA, limiting the use of MCDA in countries where HTA decision is economically driven.
	The Saved Young Life Equivalents (SAVE) approach could be useful for value assessment in very young children, considering that Person Trade-off approach from a societal perspective was applied, thus avoiding the difficulties to elicit utilities for very young children from self-perspective (8).	Although this proposal is very relevant it is unlikely to be accepted by HTA in a medium term.
Discount rate	In cases of GTs, it is proposed that differential discounting whereby the health benefits are discounted at a lower rate (e.g., 1–3.5%) than costs, and variable discounting rates that are altered over time, are more appropriate than applying a uniform and constant discounting rate for both benefits and costs (17, 22–25).	No HTA will in the short term accept to change their current discount rate for a specific class of products
	Instead of adjusting in base case scenario, it is recommended to perform sensitivity analysis including the use of varying discounting rates for benefits and costs, such as, of 0–5%, to explore the magnitude of impacts of discounting rates on ICER estimate (22, 24–26).	No HTA will in the short term accept to change their current discount rate for a specific class of products
Extrapolate method	In case of potentially curative GTs, mixture cure models allowing the incorporation of both cured and non-cured patients, may be more helpful than parametric methods to estimate the long-term survival (17, 22, 24, 26).	This would only be possible if the survival curve is not flat. Even then, it is unclear if it would be acceptable because too little is known about long-term effectiveness of GTs.
**RECOMMENDATIONS TO ADDRESS THE CHALLENGES IN AFFORDABILITY**		
Budget impact	Set a higher ICER threshold for innovative GTs considering the broader, indirect benefits it may provide (23, 25, 26, 37).	Different ICER threshold is already applied under exceptional circumstance, but it is not for the nature of the drug, but for specific condition, such as rare disease, diseases with high unmet need, or disease meeting end of life criteria.
	New approaches for value-based pricing were proposed, including sliding scale for ICER, re-pricing” of cost-offsets, QALY-based capping of value-based price and shared saving approaches (23, 26, 51).	It is unlikely that payers will officially accept such change in the pricing of such therapy, considering that it must be implemented with a radical modification of current policy
Innovative payment mechanism	Innovative payment mechanisms were proposed to facilitate the patient access to promising GTs, while at the same time to safeguard the sustainability of healthcare budgets. This generally included: financial-based payment, outcome-based payment and annuity payment (solely or in link with outcome-based payment) (19, 20, 22–26, 37, 38, 56).	In reality, the biggest issue for payers is the budget impact. The pressure of budget impact is imposed to payers at the year of administration, even if payment is based on installment. Pay-for-performance is unlikely to be appropriate as effectiveness of GTs is generally suggested for about 5 years, while such agreement is implemented by payers on short term that GTs are likely to be effective

*GTs, gene therapies; HTA, health technology assessment; ICER, incremental cost-effectiveness ratio; MCDA, multiple criteria decision analysis; NICE, national institute for health and care excellence; QALY, quality-adjusted life year*.

Following a Preferred Reporting Items for Systematic Reviews and Meta-Analyses (PRISMA) protocol, we identified articles in Medline and Embase published in English in the last 5 years that proposed any recommendations for the evidence generation for GTs as based on clinical evidence needs, economic analysis, and payer affordability considerations. The details for the search strategy and PRISMA diagram were provided in Supplementary Table 1 and Supplementary Figure 1, respectively.

## Clinical Evidence Needs

### Single-Arm Trials

Randomized controlled parallel trials (RCTs) are inarguably accepted as the gold standard approach for establishing the safety and efficacy of a new intervention (12, 13). However, RCTs for evaluating some therapies are not always feasible, especially in life-threatening or rare diseases for which therapeutic options have been exhausted (14, 15), Hence, evidence standards for regulatory approval have been made flexible to allow rapid approval of potentially effective therapies such as GTs, allowing small sample sizes or single-arm studies (16–18) being accepted to enable licensing of the treatments (19, 20).

However, such limited data have created larger-than-usual gaps for GT evidence needs (16), and strategies to improve the strength of evidence for GTs have been recommended (21). First, the inclusion of other types of non-randomized study designs could provide complementary evidence to single-arm trial data. For example, these designs could include natural history studies, observational studies, patient registry databases, or medical chart extractions to serve as historical controls (20, 22–25).

However, reducing the bias of historical control groups to complement single-arm trials must also be minimized, and the following aspects could be considered (17, 23, 26, 27): (1) the evidence suggests that the magnitude of the treatment effect size vs. the historical group is dramatic; (2) the primary endpoint is objective, durable and reproducible; (3) the heterogeneity in the patient population and study outcomes is explored and adequately adjusted with suitable statistical analysis methods (23); (4) the confounding factors are adequately adjusted with suitable statistical analysis methods; and (5) the generalizability and transferability of the clinical data toward the historical cohort were considered and discussed. In addition, network meta-analysis and multivariate meta-analysis could be used to measure comparative effectiveness and (23) reduce the uncertainty around the effect estimates obtained from the single-arm studies (20).

*DAF considerations*: Although numerous approaches to reduce payer uncertainties with single-arm studies have been published, HTA guidance on assessing evidence from single-arm trials remains scant (21). In addition, HTA bodies are more likely to accept indirect comparisons for demonstrating non-inferiority but are cautious to accept such evidence for demonstrating superiority (28). Payer uncertainties have been further fueled by the recent challenges of when data were collected in terms of the coronavirus disease 2019 (COVID-19) pandemic, which has added other confounding issues (29). Therefore, synthetic controls with high patient and outcome similarity may be more appropriate than historical controls with retrospective observational studies (30).

### Surrogate Endpoints

Due to limited long-term follow-up, the effectiveness of GTs is typically investigated on surrogate endpoints (31), especially in very rare diseases or clinically slowly progressive diseases (20). However, by having to extrapolate based on biomarker-based treatment benefits, payers are left with the uncertainty of the treatment's true clinical benefits (32). Hence, determining the appropriate approaches for the measurement and validation of surrogate endpoints for GTs concerning final clinical endpoints [e.g., mortality, survival, or health-related quality-of-life (HRQoL)] is a key consideration in HTA (22–24).

The literature has suggested that the relationships between surrogate endpoints and final clinical endpoints must be examined for: (1) biological plausibility (20, 25); (2) the association between the surrogate and the final outcome across cohorts or at the level of the individual patient (20, 25); (3) evidence that the technology improves the surrogate and the final outcome in other clinical trials, epidemiological studies or registries. In the case of GTs for rare diseases, the evidence of the same surrogate endpoints elicited from similar diseases with higher prevalence could be considered (25); (4) meta-analysis supporting the validation of surrogate endpoints to final endpoints (25); and (5) improved knowledge on the significance of biomarkers that enhance the acceptance of reliable biomarkers to demonstrate clinical benefits (23).

*DAF considerations*: Few HTA agencies currently provide guidelines on statistical methods to validate surrogate endpoints, nor provide explicit criteria to decide whether specific surrogate endpoints are more valid than others (33, 34). Furthermore, validating surrogate endpoints from natural history data from patient registries in rare conditions is particularly difficult given the limited availability of such databases (35).

### Real-World Evidence

The most critical uncertainties for GTs are the long-term effectiveness and safety. Therefore, there is a high level of consensus regarding the importance of post-launch real-world evidence (RWE) to confirm treatment benefits and bridge the evidence gaps in the initial regulatory and HTA submissions for GTs (19, 26, 32, 36–38). To enhance greater consistency and efficiency of RWE collection, coordination across countries has been encouraged (19, 23, 39). In addition, the value of information (VOI) analysis will allow assessing whether performing additional studies will provide robust added data (40).

*DAF considerations*: Depending on country-specific restrictions on reimbursement and local clinical practices, the transferability of data may be challenged. Moreover, while VOI analysis can support decision-making on the need to perform additional studies (40), it does not help HTA decision-making at the time of launch, in addition to the challenges for implementation given technical and policy-related reasons (41).

## Economic Analysis

### Valuation of Treatment Benefits

Novel economic value elements to patients, caregivers, and the whole society, that extend beyond the direct patient health benefits, are worthy of consideration when performing the value assessment of GTs. The type of treatment benefits associated with GTs ranges broadly and include clinical benefits, including quality-adjusted life-year (QALY) gains, and indirect benefits, such as the continuation of normal education, improvement of productivity, and reduction in the caring burdens of family members. In addition, GTs can bring other broader forms of value, such as scientific spillover, adherence improving factors, health equity issues, and value of cure (17, 19, 22–25, 31, 37, 42).

However, these broader values are not or insufficiently captured in the QALY and incremental cost-effectiveness ratio (ICER) calculation in current HTA reference cases. Hence, it has been argued that cost-effectiveness should provide references cases analyses from both healthcare payer and societal perspectives (17, 22, 23, 25, 26). Cost-benefit analysis has also been proposed as an option to allow capturing of all types of benefits as measured in monetary terms (17). The most commonly proposed approach for the measurements of broader values is termed “multiple criteria decision analysis” (MCDA) (22, 23, 26), which could enable the incorporation of additional values as well as their relative weight in a deliberate way.

*DAF considerations:* While numerous publications propose novel elements of value, these have had limited influence on HTA bodies which focus predominantly on clinical effectiveness and cost-effectiveness. Some broader disease-related value elements (i.e., disease severity and unmet need) are already incorporated in some agencies such as the Haute Autorité de Santé (HAS, France), Agenzia Italiana del Farmaco (AIFA, Italy), and the Spanish Agency for Evaluation of Medicines and Healthcare and the Products Interministerial Committee for Pricing (AEMPS and CIPM). Other agencies including the National Institute for Health and Care Excellence (NICE, England) and the Scottish Medical Consortium (SMC, Scotland) tend to incorporate modifiers to the ICER threshold and social cost when applicable, while Tandvårds-och läkemedelsförmånsverket (TLV, Sweden) and Zorginstituut Nederland (ZIN, The Netherlands) also consider indirect benefits (i.e., productivity improvement). However, broader societal values (i.e., scientific spill-overs and value of cure) are rarely considered (43–45). As such, it is unlikely that national HTA bodies will change their positions on the elements of value they are willing to consider. For example, despite being the output of joined EU effort, the HTA core model by EUnetHTA which considered these broader value elements (46) has hardly been implemented to date.

Moreover, the experience of the NICE to consider quantitative MCDA was negative, suggesting that flexible deliberation is preferred over stringent rules (47).

Hence, while it is very relevant for HTA to consider both payer and society perspectives of treatment benefits for an optimized resource allocation, especially for innovative and breakthrough therapies including GTs, the adoption of the societal perspectives cannot be assumed to occur in the short- or medium terms in countries where this perspective is presently not considered due to a tight administrative or time constraints (48).

### Additional Costs Beyond Acquisition Costs

Apart from the acquisition price, it has been raised that more universal costs and resources associated with GTs should be taken in the total cost calculation (22, 27). For example, these include but are not limited to additional infrastructure costs on the healthcare system (20), the cost for managing adverse events (26), and expenses for patients to travel to specialized medical centers for procedures such as for Chimeric Antigen Receptor T cell (CAR-T) treatment (22).

*DAF considerations*: A robust HTA requires that the estimations of the total cost be as accurate as possible; however, it may be difficult to capture this information due to the lack of reliable data sources for the rare diseases treated by GTs. Additionally, there remains much heterogeneity and paucity of data in cost structure, cost drivers of administration of GTs, and patients' management-related costs.

### Discounting Rates

It is argued that the discounting rates currently applied by HTA bodies are generally too high for GTs (17, 22, 23, 25) given its potential long-term benefits. Although it is common practice to apply a uniform and constant discounting rate for both benefits and costs over time (20), in cases of GTs, the justification of differential discounting has been made. This would entail health benefits to be discounted at a lower rate (e.g., 1–3.5%) than costs (22, 24), and variable discounting rates that are altered over time (22). Alternatively, sensitivity analysis is also recommended, including the use of varying discounting rates for benefits and costs (22, 24–26), such as, of 0–5%, to explore the magnitude of impacts of discounting rates on ICER estimate.

*DAF considerations*: Although a lower discounting rate (e.g., 1.5% by NICE) than normally applied has been explored for products with long-term benefits or studies with longer time horizon, such as 2.5 % when time horizon <30 years, and 1.5% thereafter in France (49), the problems identified are related to the uncertainty in the long-term benefits sufficiently meeting the defined criteria. Moreover, a declining discounting rule has been criticized for being inconsistent and unjustified (50).

In general, HTA bodies are not highly flexible on the current rules for applying a specific discount rate for a specific class of products simply afforded by its novel mechanism of action. In fact, the choice of discount rates is fundamentally subject to the local conditions and policies of the different HTA agencies, such as factors concerning public health and the economy (23, 24).

### Willingness to Pay (or ICER) Thresholds

According to the hypothesis that GTs are associated with broader, indirect values not adequately captured in the QALY estimate, some publications have recommended setting a higher ICER threshold for innovative GTs (17, 19, 23, 25, 26, 37). Other innovative approaches for value-based pricing have also been proposed, including the sliding scale for ICER, re-pricing” of cost-offsets, QALY-based capping of value-based price, and shared saving approaches (23, 26, 51).

*DAF considerations*: With higher payer willingness to pay thresholds comes the possibility that developers will be motivated to inflate the price of GTs to meet the ICER threshold (52, 53), in other words, the developers will charge the price as high as the payer are able to endure (26). It is unlikely that payers will accept innovative value-pricing methods, considering that they must be implemented with a radical modification of current policy. Obviously, GT discovery and development is flourishing, suggesting current incentives are attractive enough for developers.

### Extrapolation

Partitioned survival models, which are commonly used for the extrapolation of long-term survival for patients with cancer, have enabled early access to oncology treatments and regulatory approval based on early clinical data. However, for the economic evaluation of GTs specific to some rare diseases, partitioned survival models lack sufficient ability to reflect the patient trajectories (27), and often fail to properly incorporate uncertainty around parameter estimates (23) and complexity of diseases (22). In the case of GTs, mixture cure models allowing the incorporation of both cured and non-cured patients, have been recommended as more useful than parametric methods to estimate the long-term survival (17, 22, 24, 26).

*DAF considerations*: Extrapolations on long-term GT outcomes are only possible if efficacy shows a drop during the observation period. In most cases of GTs, the durability of effectiveness is flat, during the pre-approval period, making it impossible to extrapolate efficacy using the parametric or non-parametric methods. In addition, the adoption of new methodologies for the extrapolation of the survival curve can be a lengthy process. For example, a recent study confirmed that survival for nivolumab was underestimated using different parametric and non-parametric methods (54). However, NICE issued the guideline for flexible methods for survival analysis only after substantial evidence has accumulated to prove that standard parametric extrapolation is not fitted for treatments with a delayed response, such as immunotherapy (55). Long-term accumulation of robust evidence will nonetheless shift the current resistance of HTA to consider non-conventional extrapolation methods.

## Affordability

### Budget Impact and Innovative Payment Mechanisms

Despite the measured cost-effectiveness of GTs, the impact of their high upfront cost on payer budgets remain (17, 19, 25, 26). Therefore, innovative payment mechanisms have been proposed to facilitate patient access to promising GTs, meanwhile safeguarding the sustainability of healthcare budgets. Such mechanisms have mainly comprised: financial-based payment (19, 22–24, 26, 56). including simple discount, rebates, volume-based pricing, “Netflix subscription” model, funding-based payment, re-insurance, and healthcare loans; outcome-based payment (19, 22, 23, 25, 26, 37, 38, 56), including pay-for-performance and risk-sharing agreements; and annuity or installment payment (solely or in link with outcome-based payment) (20, 22, 23, 26, 56) and amortization (19, 57).

*DAF considerations*: Generally evidenced for about 5 years, pay-for-performance agreements cannot be practical mechanisms for funding GTs, given the inadequate window of time to assess the effectiveness of the treatment. Thus, the ability of pay-for-performance mechanisms to account for the long-term uncertainty associated with GTs is limited. In addition, the pressure of budget impact is imposed on payers at the year of administration, even if payment is based on installment. Indeed, generally accepted accounting principles require that a consumable (such as a drug) is accounted for on the balance sheet within the year of acquisition. However, the key accounting principle of amortization may offer a new approach for healthcare payers to unlock access to GTs while spreading the budget impact over several years (58).

## Discussion

Our review has summarized several approaches for HTA evidence generation to meet the idiosyncrasies of GTs. While these theoretical perspectives are rational and necessary, from a pragmatic perspective, much flexibility is necessary from HTA bodies to put recommendations into practice. Yet, the flexibility of tailoring current DAFs within HTA organizations is not a likely reality in the near term.

While a new class of product with a novel mechanism of action may stimulate arguments that a specific reference case is necessary, mainstream opinions indicate that adjustments could be considered for GTs, although a new reference case is not needed (25). For example, NICE has delivered systematically negative decisions for most innovative oncology products until the end-of-life criteria tailor-made for oncology was introduced several years later, which enabled a higher ICER threshold to be acceptable for products satisfying the pre-defined standards. Other initiatives included Cancer Drug Fund was introduced by NHS England to temporarily reimburse oncology drugs with promising clinical benefits but associated with significant uncertainties, and a highly specialized technology (HST) pathway to evaluate the expensive drugs indicated for a distinct group of diseases, such as ultra-rare conditions. These examples suggest how conservative HTA bodies can be in modifying their DAF or their reference case.

Hence, given all the evidence needed for GTs, to ensure fair and timely evolution of DAF of HTA proactive interactions with relevant stakeholders could be powerful and well-warranted (19, 38), and to include patients, clinicians, experts (e.g., medical scientists, statisticians, and pharmacoeconomics professionals), industry representatives, regulators, and HTA bodies. Such collaboration will allow more knowledge on the varying interests and expectations of each party (36), and also offer advantages to foster a better understanding of the scientific mechanisms of the interventions (23), to increase the reliability of the estimates on magnitude and durability of treatment benefits (17). Recently a checklist was published providing a pragmatic approach that could easily be adopted by HTA (25). In the absence of such an initiative, scientific publications and recommendations continue to accumulate on this topic; although important from an academic perspective, they will have limited impacts on GT HTA.

## Conclusion

Published recommendations for evidence generation and economic assessment of GTs currently appear to have limited impact on HTA DAF due to either them being more theoretical in orientation rather than being pragmatic or because of conservative attitudes of HTA on DAF. Multi-stakeholders dialogue is warranted to enhance communications that will allow more certain and rapid assessments of GTs, thus enabling optimized patient access at a reasonable budget impact.

## Data Availability Statement

The original contributions presented in the study are included in the article/Supplementary Material, further inquiries can be directed to the corresponding author/s.

## Author Contributions

MT conceived the design of this review, drafted the table of contents, and provided expert insights. TQ and GS wrote the entire manuscript. MP, SL, EP, and JJ contributed to the literature searches and abstract drafting. All authors contributed to the article and approved the submitted version.

## Conflict of Interest

MP, EP, JJ, and MT was employed by the company Creativ-Ceutical. GS was employed by company Apothecom. The remaining authors declare that the research was conducted in the absence of any commercial or financial relationships that could be construed as a potential conflict of interest.

## Publisher's Note

All claims expressed in this article are solely those of the authors and do not necessarily represent those of their affiliated organizations, or those of the publisher, the editors and the reviewers. Any product that may be evaluated in this article, or claim that may be made by its manufacturer, is not guaranteed or endorsed by the publisher.
