# Supplementary material for: Gene Therapy Evidence Generation and Economic Analysis: Pragmatic Considerations to Facilitate Fit-for-Purpose Health Technology Assessment
Source: Front Public Health. 2022 Feb 9;10:773629. doi: 10.3389/fpubh.2022.773629 (PMC8863657; doi:10.3389/fpubh.2022.773629)
Supplement: Supplementary file 1 [file Data_Sheet_1.docx]

# Supplementary Materials

## Table 1. Keywords for literature search

| **Keywords for gene therapies** | \| gene therapy \| \| --- \| \| regenerative medicine \| \| curative \| \| advanced therapeutic medicinal product \| \| replacement therapy \| \| gene editing \| \| T-cell therapy \| \| innovative medicine \| \| life-extending \| \| life-threatening \| \| gene therapies \| \| gene therapy \| \| regenerative medicine \| \| advanced therapeutic medicinal product \| \| replacement therapy \| \| T-cell therapy \| \| innovative medicine \| \| gene therapies \| \| innovative \| \| gene replacement therapy \| \| advanced therapies \| \| chimeric antigen receptor T-cell therapy \| \| Alipogene tiparvovec \| \| talimogene laherparepvec \| \| voretigene neparvovec-rzyl \| \| tisagenlecleucel \| \| axicabtagene ciloleucel \| \| brexucabtagene autoleucel \| \| onasemnogene abeparvovec-xioi \| \| betibeglogene autotemcel \| \| betibeglogene autotemcel \| \| voretigene neparvovec \| \| Autologe CD34+-angereicherte Zellfraktion \| \| Imlygic \| \| Zalmoxis \| \| Strimvelis \| \| Luxturna \| \| Kymriah \| \| TECARTUS \| \| Yescarta \| \| ZOLGENSMA \| \| Zynteglo \| \| Glybera \| \| Gene \| \| Gendicine \| \| Oncorine \| |
| --- | --- | --- | --- | --- | --- | --- | --- | --- | --- | --- | --- | --- | --- | --- | --- | --- | --- | --- | --- | --- | --- | --- | --- | --- | --- | --- | --- | --- | --- | --- | --- | --- | --- | --- | --- | --- | --- | --- | --- | --- | --- | --- | --- | --- | --- | --- | --- |
| **Keywords for study types** | cost-effectiveness  cost-utility  cost-benefit  economic evaluation  Pharmacoeconomics  health technology assessment  methodological recommendation  cost-effectiveness analyses  cost-utility analyses  cost-benefit analyses  economic evaluation  Pharmacoeconomics  health technology assessment  methodological recommendation  Pharmacoeconomic |

## Figure 1. PRISMA flow diagram for the literature selection

Full-text articles excluded, with reasons
(n =104)

Full-text articles assessed for eligibility
(n =122)

Studies included in qualitative synthesis
(n =18)

Records excluded
(n =2297)

Records with title and abstracts screened
(n = 2670)

Records after duplicates removed
(n =2419)

## Inclusion

## Eligibility

## Screening

Records identified through database searching
(n = 2458)

## Identification
